# Clinical Pharmacokinetics and Dose Recommendations for Posaconazole in Infants and Children

**DOI:** 10.1007/s40262-018-0658-1

**Published:** 2018-04-20

**Authors:** Sophida Boonsathorn, Iek Cheng, Frank Kloprogge, Carlos Alonso, Charmion Lee, Bilyana Doncheva, John Booth, Robert Chiesa, Adam Irwin, Joseph F. Standing

**Affiliations:** 1 Infection, Inflammation, Immunity Section, Room 661, UCL Great Ormond Street Institute of Child Health, University College London, 30 Guilford Street, London, WC1N 1EH UK; 2 Ramathibodi Hospital, Mahidol University, Bangkok, Thailand; 3 Great Ormond Street Hospital for Children, London, UK; 4 Institute of Global Health, University College London, London, UK; 5 University of Queensland Centre for Clinical Research, Brisbane, QLD Australia; 6 Paediatric Infectious Diseases Research Group, St. George’s, University of London, London, UK

## Abstract

**Objectives:**

The objectives of this study were to investigate the population pharmacokinetics of posaconazole in immunocompromised children, evaluate the influence of patient characteristics on posaconazole exposure and perform simulations to recommend optimal starting doses.

**Methods:**

Posaconazole plasma concentrations from paediatric patients undergoing therapeutic drug monitoring were extracted from a tertiary paediatric hospital database. These were merged with covariates collected from electronic sources and case-note reviews. An allometrically scaled population-pharmacokinetic model was developed to investigate the effect of tablet and suspension relative bioavailability, nonlinear bioavailability of suspension, followed by a step-wise covariate model building exercise to identify other important sources of variability.

**Results:**

A total of 338 posaconazole plasma concentrations samples were taken from 117 children aged 5 months to 18 years. A one-compartment model was used, with tablet apparent clearance standardised to a 70-kg individual of 15 L/h. Suspension was found to have decreasing bioavailability with increasing dose; the estimated suspension dose to yield half the tablet bioavailability was 99 mg/m^2^. Diarrhoea and proton pump inhibitors were also associated with reduced suspension bioavailability.

**Conclusions:**

In the largest population-pharmacokinetic study to date in children, we have found similar covariate effects to those seen in adults, but low bioavailability of suspension in patients with diarrhoea or those taking concurrent proton pump inhibitors, which may in particular limit the use of posaconazole in these patients.

**Electronic supplementary material:**

The online version of this article (10.1007/s40262-018-0658-1) contains supplementary material, which is available to authorized users.

## Key Points

**Table:** 

Posaconazole is unlicensed for children under 13 years of age and its pharmacokinetics have not widely been reported in this population group; our study provides a large cohort in this age group receiving both tablets and an oral suspension
A population-pharmacokinetic model has revealed saturable suspension bioavailability, and reduced bioavailability in patients taking proton pump inhibitors and those with diarrhoea
Based on simulations from our model, dosing and therapeutic drug monitoring guidelines are provided

## Introduction

Invasive fungal disease (IFD) remains an important cause of morbidity and mortality in immunocompromised children [1, 2]. Despite the development of new diagnostic methods and the availability of new antifungal agents, the incidence and mortality from IFD remains unacceptably high. Posaconazole is a second-generation, broad-spectrum, fluorinate triazole that inhibits ergosterol synthesis in the fungal cell wall. It is active against most pathogenic yeasts and moulds, including *Aspergillus* spp., *Candida* spp., *Cryptococcus* spp., filamentous fungi, dimorphic fungi and endemic mycoses [3–6].

Despite currently being unlicensed for use in the paediatric population, posaconazole has successfully been used for the prevention and treatment of IFD in this group [7], and is recommended for prophylaxis against invasive Aspergillus and Candida infections after allogeneic haematopoietic stem cell transplantation in adolescents [7]. Additionally, posaconazole has been used as a salvage treatment for IFD with favourable outcomes [8, 9].

Two oral formulations of posaconazole are currently available, a gastro-resistant tablet and an oral suspension. Posaconazole pharmacokinetics are variable, particularly during absorption and with the suspension formulation, and very limited paediatric data have been published to date [10]. Pharmacokinetic models to inform optimal dosing in infants and young children, in particular, are therefore lacking.

Therapeutic drug monitoring (TDM) for most triazoles is recommended owing to high inter-individual variability and the potential for drug–drug interactions. According to the British Society for Medical Mycology, a posaconazole target trough concentration of greater than 0.7 and 1 mg/L should be used for the prophylaxis and treatment of IFD, respectively, and as yet no upper limit for toxicity has been defined [11].

Our study aimed to develop a population-pharmacokinetic model of posaconazole in a large cohort of paediatric patients. Focussing on children aged 12 years and under, the resulting model was then used to identify patient groups at risk of sub-optimal posaconazole exposure, and to suggest initial dosing.

## Patients and Methods

### Patients and Data Collection

In- and out-patients at a tertiary paediatric hospital receiving posaconazole between January 2010 and December 2016 were studied. Patients receiving posaconazole for prophylaxis or the treatment of IFD and who had at least one TDM sample taken, and had full dosing and sample timing history available were included. The time and date of the posaconazole TDM sample, along with the reported concentration, were extracted from electronic TDM records. For inpatients, dosing history was taken from electronic nursing administration history, whereas for outpatients the time of the last dose was taken from the TDM request. In addition, demographics, concomitant medications, presence of diarrhoea on the day of sampling and purpose (prophylaxis or treatment) were collected from electronic records. Medical notes including clinic letters and inpatient treatment records coinciding with each sampling occasion were read to extract information on the indication and the presence of diarrhoea. Because the data were collected by clinical staff retrospectively and were anonymised prior to analysis, ethical review and the need for informed consent were waived by the institute’s research and development office.

For dosing data from the electronic prescribing and administration system, all doses from the first dose to the first TDM sample were included. Thereafter, only the doses in the preceding 48 h prior to a TDM sample were used, with the first of these assumed to be at steady state. For outpatient samples, the preceding dose was assumed to be at steady state based on the reported dose and frequency.

During the recruitment period, posaconazole assays were sent to the following accredited laboratories for analysis: Department of Microbiology, Wythenshawe Hospital, Manchester, UK; the Mycology Reference Laboratory, Leeds, UK; and Mycology Reference Laboratory, Bristol, UK. The lower limits of quantification ranged between 0.07 and 0.2 mg/L.

### Population-Pharmacokinetic Modelling

Because most samples were pre-dose troughs and posaconazole is known to have a long elimination half-life, a one-compartment model with first-order absorption was used. Allometric scaling with exponents of 0.75, 1 and − 0.25 on clearance (CL), central volume and absorption rate constant (Ka) were added a priori, and a sigmoidal maturation function based on postmenstrual age was tested [12].

Because posaconazole tablets have been reported to have higher bioavailability than the suspension [13], and tablet pharmacokinetics are linear in the therapeutic range [14], whereas suspension has been shown to have nonlinear absorption [15], the following expression was used to describe relative bioavailability between a tablet and a suspension, and the nonlinear suspension bioavailability:

$$F = F_{\text{tab}} - \frac{D}{{D + \beta_{\text{dose}} }},$$

where $F$ is the bioavailability of the suspension relative to the tablet, $F_{\text{tab}}$ is the apparent tablet bioavailability that was fixed to 1, $D$ is the dose in mg/m^2^, and $\beta_{\text{dose}}$ is the estimated dose in mg/m^2^ to yield a 50% decrease in bioavailability of the suspension relative to tablets.

A step-wise covariate model (SCM) building exercise with a forward inclusion limit set to a *p* value of 0.05 and backwards elimination limit set to a *p* value of 0.01 was then undertaken to identify whether any of the following dichotomous covariates were associated with suspension apparent bioavailability: diarrhoea, treatment/prophylaxis, macrolides, echinocandins, terbinafine, ciclosporin, tacrolimus, mycophenolate, rifamycins, carbamazepine, phenytoin, histamine H_2_-receptor antagonists, proton pump inhibitors (PPIs) or valaciclovir. The following concomitant medications were also tested on CL: macrolides, echinocandins, ciclosporin, tacrolimus, mycophenolate, rifampicin, carbamazepine, phenytoin or valaciclovir.

Model diagnostics included plots of observations vs. population predictions and conditional weighted residuals vs. time and prediction. Simulation properties were tested with a visual predictive check. Parameter stability was investigated using a non-parametric bootstrap. Modelling was undertaken using NONMEM Version 7.3 (ICON PLC, Dublin, Ireland) [16] with the first-order conditional estimation algorithm with interaction. A combined additive plus proportional error model was used throughout model building, and then removal of the additive or proportional element considered at the final model step.

A decrease in − 2 log likelihood [the objective function value (OFV) in NONMEM] between two nested models asymptotically follows a $\chi^{2}$ distribution with degrees of freedom equal to the number of additional parameters. This was used to guide covariate inclusion with a *p* value threshold set to 0.01. Perl-speaks NONMEM (University of Uppsala, Sweden) was used for the SCM (forward inclusion *p* < 0.05, backward elimination *p* < 0.01), visual predictive check and bootstrap preparation [17], and data manipulations and plotting were performed using R Version 3.2 (R Foundation, Vienna, Austria) [18].

A dataset of 1000 hypothetical patients for each significant covariate in the final model and for each formulation was created by re-sampling from the demographics (weight, age) of the original dataset. Using this dataset and the final model, simulations of steady-state trough concentration were produced to assess probability of target attainment for prophylaxis (0.7 mg/L) and treatment (1 mg/L) targets [11].

## Results

### Data Characteristics

**Fig. 1:**
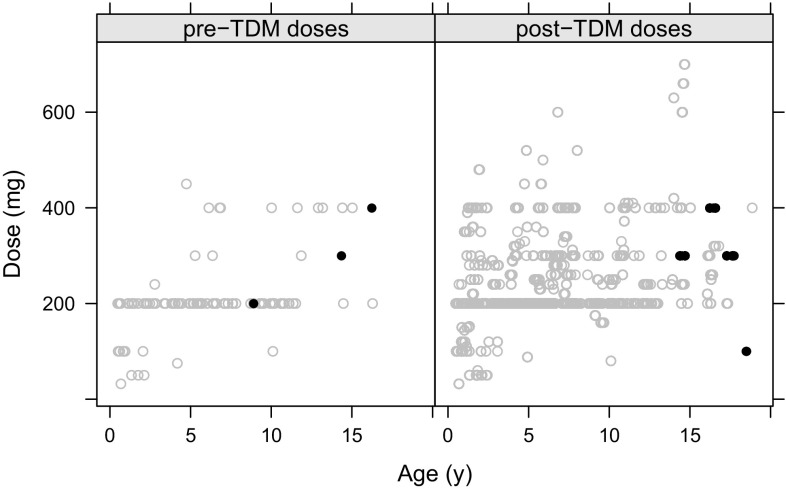
Absolute dose (in milligrams) administered vs. age (in years). The left-hand plot shows initial dosing prior to therapeutic drug monitoring (TDM) sampling and the right-hand plot shows doses administered after at least one TDM sample. Grey circles represent suspension doses and black filled points represent tablets

The initial dataset contained 580 posaconazole plasma concentrations from 128 individuals. Owing to incomplete data entry in outpatient TDM records that could not be reconciled from clinic letters, 242 samples were excluded, leaving a total of 338 posaconazole plasma concentrations from 117 children. There were 22 samples below the limit of quantification, which were substituted with a value of lower limit of quantification/2. Most samples were taken following administration of a posaconazole suspension (326). The median age was 5.7 years, which included 22 infants aged less than 2 years, 47 young children aged 2–6 years and 36 children aged 7–12 years. Demographic details are given in Table 1, and an illustration of doses administered by age is given in Fig. 1.

**Table 1:** Demographics of all patients, and those included in the pharmacokinetic analysis after removing data with missing dose history or sample timing information

Variable	All patients (*n* = 128)	PK patients (*n* = 117)
No. of TDM samples	580	338
No. of samples/patients, median (range)	5 (1–14)	3 (1–11)
Speciality, BMT/Haem/Imm/other^a^	94/7/19/8	87/6/17/7
Age, years (range)	5.9 (0.5–18.9)	5.7 (0.5–18.5)
Weight, kg (range)	17.92 (6.05–71)	17.8 (6.05–74.8)
Sex, male/female	47/81	43/74
Dose, mg (range)	200 (32–700)	200 (32–630)
Dose, mg/kg (range)	12.99 (2.58–48.95)	13.11 (2.67–48.95)
Dose, mg/m^2^, (range)	326 (84–921)	326 (84–921)
Concentration, mg/L (range)	0.96 (0.07–4.99)	0.8 (0.07–4.99)
Sample time after dose, h(range)	6.96 (0.02–24.78)	6.52 (0.02–24.78)
Dose frequency, doses/day (range)	3 (1–4)	3 (1–4)
% samples when patient had diarrhoea	18	20
% samples when patient also taking PPI	61	68
% samples when patient also taking H_2_ receptor antagonist	28	32

*BMT* bone marrow transplant, *Haem* haematology, *Imm* immumology, *H*_*2*_ histamine H_2_-receptor antagonist, *PK* pharmacokinetic, *PPI* proton pump inhibitor, *TDM* therapeutic drug monitoring

^a^‘Other’ includes patients undergoing solid organ transplantation, those from gastroenterology, and surgical patients

### Population Pharmacokinetics

The base structural model with allometric scaling centred on 70 kg and inter-individual variability on CL only gave parameter estimates of 86.5 L/h, 1439.6 L and 0.09/h for apparent clearance (CL/F), apparent volume and Ka, respectively. The addition of a sigmoidal maturation function gave no improvement in fit, whereas adding a categorical covariate of a change in relative bioavailability with suspension compared with a tablet yielded a decrease in OFV of 10.11 (*p* = 0.0015). Allowing suspension bioavailability relative to a tablet to change with dose ($\beta_{\text{dose}}$ in the expression above) yielded a decrease in OFV of 23 (*p* < 0.001) compared with the model of suspension having a fixed decrease in bioavailability regardless of dose.

During model building, flip-flop kinetics became apparent (Ka being estimated to be larger than elimination rate constant). Thereafter, Ka was fixed to literature values of 0.588/h for tablets [19], and 0.197/h for suspensions [15]. The difference in OFV between this model and the estimated Ka and V models was 7.63, indicating a very similar fit.

**Fig. 2:**
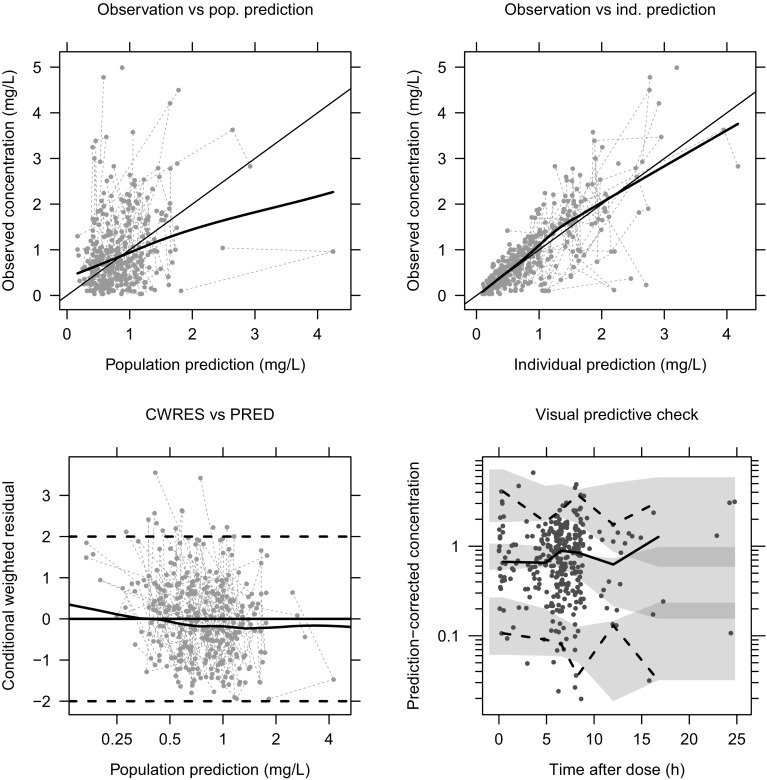
Goodness-of-fit plots for the final model. Top row: population (pop.) predictions vs. observations, individual (ind.) predictions vs. observations. Bottom row: conditional weighted residuals (CWRES) vs. pop. prediction (PRED), and PRED-corrected visual predictive check showing model-simulated 95% confidence intervals for the simulated 2.5, 50 and 97.5th percentiles (shaded areas) compared with observed percentiles (lines)

In the SCM, following the backward elimination step, diarrhoea and concurrent PPI administration both resulted in significant improvements in fit when applied to a decrease in suspension bioavailability ($\Delta$OFV 11.06 and 35.53 corresponding to *p* = 0.001 and *p* < 0.001, respectively). Parameter estimates and bootstrap results are given in Table 2. Figure 2 shows goodness-of fit plots for the final model.

**Table 2:** Parameter estimates from the final model (the NONMEM model code is given in the Electronic Supplementary Material)

Parameter	Estimate (%RSE)	IIV %CV (%RSE)	Bootstrap median (95% CI)	Bootstrap IIV %CV (95% CI)
CL/F, L/h	14.95 (34.5)	63 (23.9)	14.6 (6.3–34.1)	63 (49–79)
V/F, L	201.7 (38.8)	–	213 (80.7–904.3)	–
Ka suspension/h	0.197 (fixed)	–	–	–
Ka tablet/h	0.588 (fixed)	–	–	–
*β*_dose_, mg/m^2^	99 (44.4)	–	97.6 (36.5–341.7 )	–
*θ*_D_	−0.33 (28)	–	−0.32 (−0.52 to −0.13)	–
*θ*_P_	−0.42 (14.9)	–	−0.42 (−0.53 to −0.27)	–
Proportional error, %CV	47.29 (0.2)	–	46.43 (36.92–53.48)	–
Additive error, mg/L	0.02 (82.7)	–	0.01 (0.001–0.07)	–

$\beta_{dose}$ estimated dose in mg/m^2^ for suspension bioavailability to drop to half that of the tablet, *CI* confidence interval, *CL/F* apparent clearance, *CV* coefficient of variation, *Ka* absorption rate constant, $\theta_{D}$ fractional decrease in suspension bioavailability with patients with diarrhea, $\theta_{P}$ fractional decrease in suspension bioavailability with patients taking proton pump inhibitors, *RSE* relative standard error, *V/F* apparent volume

### Simulations of Steady-State Trough Concentration

Simulations showed that patients aged 7–12 years taking 300 mg in a tablet form once a day (the adult treatment dose) would have a 24% probability of achieving a trough concentration of > 1 mg/L and a 33% of achieving trough concentrations of > 0.7 mg/L for prophylaxis. Giving the same total daily dose split three times per day would achieve a probability of target attainment (PTA) of 44 and 59% for treatment and prophylaxis, respectively, whereas 200 mg three times per day would achieve a PTA of 72 and 80% for treatment and prophylaxis, respectively.

**Fig. 3:**
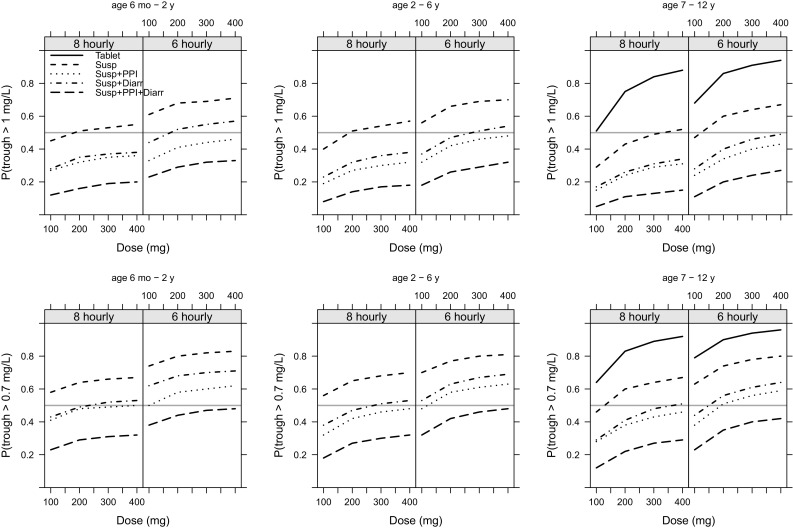
Simulated probability of trough concentration being >1 mg/L (top row) for treatment or >0.7 mg/L (bottom row) for 8- and 6-h dosing split by age group. The solid line represents tablets, the dashed line represents the suspension, the dotted line represents patients taking the suspension also receiving proton pump inhibitors, the dot/dashed line represents patients receiving the suspension who also had diarrhoea, and the long dashed line represents patients receiving the suspension and proton pump inhibitors and who had diarrhoea. The grey horizontal line represents a 50% probability of target attainment

Suspension bioavailability affected the PTA markedly, in particular in patients with diarrhoea and those taking PPIs. For example, a child aged between 6 months and 2 years taking 200 mg four times per day would have a PTA of 68 and 80% for treatment and prophylaxis, respectively, but this falls to 29 and 44% for patients with diarrhoea and those taking PPIs. In this case, doubling the dose to 400 mg four times per day only improves the PTA to 33 and 48%. Plots of simulated target attainment vs. dose are given in Fig. 3.

## Discussion

To the best of our knowledge, this is the first population-pharmacokinetic analysis of posaconazole tablets and suspension in immunocompromised children. We studied the pharmacokinetics in 117 patients, including 105 aged under 13 years. This is a larger cohort than the largest published adult clinical cohort to date by Dolton et al. [20], who studied 102 patients. Our major finding is that as soon as children are able to swallow whole tablets, they should be given the tablet formulation because a poor and saturable suspension bioavailability, particularly in patients with diarrhoea or those taking concurrent PPI therapy, means a therapeutic target attainment with suspension may be as low as 30% even on the highest feasible dose (Fig. 3).

A one-compartment model with inter-individual variability best described the pharmacokinetics of posaconazole in this study (Fig. 2). This was consistent with previous adult models [15, 19, 20]. Our estimated CL/F and apparent volume related to the tablet formulation and standardised to a 70-kg individual were 14.95 L/h and 201.68 L, respectively. In a recent study of adults, CL/F and apparent volume were estimated to be 7.3 L/h and 420 L, respectively [19], which fall within the 95% confidence intervals of our estimates (Table 2). Fixing the absorption rate to that previously reported in adults had a negligible effect on model fit, and the flat profiles, the fact our data did not include any patients sampled after their first dose, and the limited number of samples in the absorption phase all account for this. The residual variability was rather high in our study (Table 2), reflecting the fact that these were observational TDM data on a drug with highly variable pharmacokinetics. However, model diagnostics show a reasonable fit (Fig. 2). We did not find a significant relationship between age and posaconazole CL/F, which could be owing to the fact that our youngest patient was 6 months old, whereas rapid pharmacokinetic maturation tends to occur in the neonatal to early infant age group [12].

The gastro-resistant tablet formulation has been widely reported to have improved bioavailability over the suspension [21–24]. In addition, suspension bioavailability has previously been reported to be saturable in adults [25–28], although we are not aware of this relationship having previously been modelled using the population approach. In children, we found a dose–proportional relationship with our estimated dose to reach a 50% relative bioavailability reduction in tablets relative to the suspension of 99 mg/m^2^. In this expression, we scaled dose by body surface area under the assumption that gastrointestinal surface area and body surface area would be correlated, and that gastrointestinal surface area is important for absorption. Validating this assumption is not straight-forward because accurate measurement of gastrointestinal surface area is difficult [29], and no extensive studies appear to have been conducted on how it might scale with age [30]. However, our model did provide an adequate fit to our data and our estimate ought to be robust because we studied a large dose range (Table 1).

Gastrointestinal complications are common in cancer patients and haematopoietic stem cell transplantation recipients. In this analysis, 20% of patients had diarrhoea during treatment, and the majority were receiving concomitant acid suppression therapy (Table 1). Diarrhoea results in increased gastric emptying with reduced gastrointestinal residence time. This disruption in gastrointestinal function was associated with a significant reduction in bioavailability and therefore target attainment (Fig. 3). The association of diarrhoea with decreased posaconazole exposure has previously been noted in adults [20, 31], with Dolton et al. [20] finding a 45% reduction in apparent bioavailability. Our estimate of 33% shows a similar relationship in children.

Concomitant use of PPIs was associated with a 42% reduction in relative bioavailability. Concomitant PPI therapy has been shown to be associated with decreased bioavailability in adults [32–35], and our estimate is similar to that obtained by Dolton et al. [20], who found a 45% decrease. In contrast with PPIs, fewer studies have shown the potential effect of histamine H_2_-receptor antagonists on posaconazole exposure [20, 31], and we also did not find this effect, suggesting the more potent acid suppression of PPIs [36, 37] limits posaconazole absorption. It is unlikely that this interaction is cytochrome P450 mediated because posaconazole undergoes limited metabolism primarily by UDP-glucuronosyltransferase UGT1A4 [38]. Information on whether the dose was taken with food and whether mucositis was present was unavailable in our study but these may also have been significant covariates based on adult experience [20]. Partly because of the low number of children in our data taking tablets, and also the fact that PPIs and histamine H_2_-receptor antagonists have been shown not to affect posaconazole tablet bioavailability in adults [39], we did not perform covariate analysis on the tablet formulation.

Patients undergoing haematopoietic stem cell transplantation usually require immunosuppressive agents for the prevention and treatment of graft vs. host disease in combination with antifungal prophylaxis for IFD. Concurrent use of posaconazole potentially results in increased drug exposure of several immunosuppressive drugs including ciclosporin, tacrolimus, sirolimus and everolimus [40–42]. We did not find these agents to affect posaconazole pharmacokinetics, but future work on our data to investigate and quantify the effect of posaconazole on immunosuppressant levels is planned. We did not find phase II glucuronide enzyme inducers such as rifampicin or phenytoin to be significantly associated with either posaconazole CL/F or bioavailability during the SCM. The likely explanation for this is that our study contained a small proportion of samples taken concurrently with these drugs (5 and 1%, respectively), but it is also possible that immaturity of drug-metabolising enzyme expression in younger children means such interactions are less pronounced. We also tested prophylaxis vs. treatment as a covariate with the concern that the differences seen may be owing to data inaccuracies because prophylaxis patients were more likely to be outpatients with less reliable dosing history than inpatients for whom we had electronic administration data. The fact that this did not emerge as a covariate on CL/F or bioavailability indicates no such bias was present.

In Fig. 1, we show the dosing by age split between initial dose and post-TDM dosing. The key features of this plot are that a flat 200-mg dose was often used, regardless of age, and the following TDM doses were generally increased, particularly in younger patients. Clinical practice has evolved in our centre from weight-scaled dosing to fixed 200-mg dosing regardless of age, based on repeated failures to achieve therapeutic target trough concentrations. The added insight provided by simulations from our model (Fig. 3) indicates that absolute dose increases above 200 mg are rather futile owing to the saturable bioavailability. For example, a 1-year-old individual with a body surface area of 0.5 m^2^ receiving 100, 200 or 400 mg of suspension will have a relative bioavailability of 0.33, 0.2 or 0.11, respectively. Increasing from 100 to 200 mg decreases the bioavailability by 40%, whereas increasing from 200 to 400 mg decreases the bioavailability by almost half, explaining the marginal increases in trough concentration with increasing doses. In common with findings for itraconazole [43], increasing the frequency is more successful, but dose administrations of greater than four times per day are simply impractical.

Whilst we have modelled the largest paediatric posaconazole pharmacokinetics dataset to date, our study does have limitations that should be considered when interpreting the results. First, as mentioned above, these were retrospective TDM data collected over 7 years in a single centre, and owing to inconsistent reporting of the sample time and dose time in the outpatient data, we had to exclude 242 samples. Furthermore, we are likely to have collected more data on patients with poor target attainment because those patients would be sampled more frequently following dose escalation. Ideally, we would have run a prospective study with optimally designed pharmacokinetic sampling [44], but this would have resulted in a smaller dataset and then potentially missing covariates of interest. Having said this, maximum likelihood methods should not be biased by this type of data and our prediction-corrected visual predictive check showed good agreement with observations. Further data pooling experience from multiple centres would however be useful to confirm our findings. We have also performed simulations aiming for trough concentration targets based on adult data [11], whereas either a different target or use of a metric such as the area under the curve may be more appropriate in children. Whilst we did not collect outcome data during this particular study, there is a clear need for such data in this population.

## Conclusion

Our study illustrates the challenge of achieving therapeutic posaconazole trough concentrations, particularly in infants and young children administered a posaconazole suspension. In a child with diarrhoea and concomitant PPI use, therapeutic targets are unlikely to be reached in a large proportion of patients, with a low probability of target attainment with any feasible dose (Table 3).

**Table 3:** Recommended initial dosing for treatment and prophylaxis, and suggested dose adjustment following therapeutic drug monitoring (TDM)

Age group, years	Initial treatment dose	Treatment dose increase if TDM < 1 mg/L	Initial prophylaxis dose	Prophylaxis dose increase if TDM < 0.7 mg/L
6 months to < 2	200-mg suspension 4 times per day	Little value in dose increase	200-mg suspension 3 times per day	200-mg suspension 4 times per day
2–6	200-mg suspension 4 times per day	Consider increase to 300 mg 4 times per day	200-mg suspension 3 times per day	200-mg suspension 4 times per day
7–12, cannot take tablets	300-mg suspension 4 times per day	400-mg suspension 4 times per day	300-mg suspension 3 times per day	300-mg suspension 4 times per day
7–12, can take tablets	200-mg tablet 3 times per day	200- to 300-mg tablet 4 times per day	200-mg tablet 3 times per day	300-mg tablet 3 times per day

Therefore, there is an urgent need first for the intravenous formulation to be studied for IFD treatment in this population, and second, for therapeutic targets to be studied in this population to ascertain whether treatment and prophylactic benefits maybe retained with trough concentrations below 1 or 0.7 mg/L.

## Electronic supplementary material

Below is the link to the electronic supplementary material.

Supplementary material 1 (PDF 131 kb)
