# Correction to: Clinical Pharmacokinetics and Dose Recommendations for Posaconazole in Infants and Children

**DOI:** 10.1007/s40262-018-0722-x

**Published:** 2018-12-17

**Authors:** Sophida Boonsathorn, Iek Cheng, Frank Kloprogge, Carlos Alonso, Charmion Lee, Bilyana Doncheva, John Booth, Robert Chiesa, Adam Irwin, Joseph F. Standing

**Affiliations:** 1 Infection, Inflammation, Immunity Section, Room 661, UCL Great Ormond Street Institute of Child Health, University College London, London, WC1N 1EH UK; 2 Ramathibodi Hospital, Mahidol University, Bangkok, Thailand; 3 Great Ormond Street Hospital for Children, London, UK; 4 Institute of Global Health, University College London, London, UK; 5 University of Queensland Centre for Clinical Research, Brisbane, QLD Australia; 6 Paediatric Infectious Diseases Research Group, St. George’s, University of London, London, UK

## Correction to: Clin Pharmacokinet 10.1007/s40262-018-0658-1

The Open Access license, which previously read:

**Open Access** This article is distributed under the terms of the Creative Commons Attribution-NonCommercial 4.0 International License (http://creativecommons.org/licenses/by-nc/4.0/), which permits any noncommercial use, distribution, and reproduction in any medium, provided you give appropriate credit to the original author(s) and the source, provide a link to the Creative Commons license, and indicate if changes were made.

Should read:

**Open Access** This article is distributed under the terms of the Creative Commons Attribution 4.0 International License (http://creativecommons.org/licenses/by/4.0/), which permits unrestricted use, distribution, and reproduction in any medium, provided you give appropriate credit to the original author(s) and the source, provide a link to the Creative Commons license, and indicate if changes were made.

