# Supplementary material for: Clinical Pharmacokinetics and Dose Recommendations for Posaconazole in Infants and Children
Source: Clin Pharmacokinet. 2018 Apr 20;58(1):53–61. doi: 10.1007/s40262-018-0658-1 (PMC6326087; doi:10.1007/s40262-018-0658-1)
Supplement: Supplementary file 1 — Supplementary material 1 (PDF 131 kb) [file 40262_2018_658_MOESM1_ESM.pdf]

SUPPLEMENTARY MATERIAL: Clinical Pharmacokinetics and Dose Recommendations for Posaconazole in Infants and Children

1 NONMEM model file with values set to final estimates

```
$PROBLEM    Posaconazole Model
$INPUT      IDORIG ID AGE TIME TAD AMT EVID DV LNDV CMT FORM FREQ II
            SS JAC SEX WT MISSLVL MISSDOST MISSDOS MACROLIDES
            ECHINOCANDINS TERB PI CIC FK MMF RIF CBZ PHT H2 PPI ART
            VAL VERAPQUIN MISSFREQ MISSFORM MISSWT DIARRHOEA
            PROPHYLAXIS BLQ BSA LOQ SUSP NSAMP ONESAMP
$DATA      posaconazole_GOSH_data_171120.csv IGNORE=I
$SUBROUTINE ADVAN2 TRANS1
$PK

TVCL    = THETA(1) * (WT/70)**0.75
CL      = TVCL * EXP(ETA(1))

TVVC    = THETA(2) * (WT/70)
VC      = TVVC

IF(FORM==1) TVKA = THETA(3) * (WT/70)**(-0.25)
IF(FORM==2) TVKA = THETA(4) * (WT/70)**(-0.25)
KA      = TVKA
;Diarrhoea effect on susp only
IF(DIARRHOEA==1) F1DIARRHOEA = 1  ; No diarrhoea
IF(DIARRHOEA==2) F1DIARRHOEA = ( 1 + THETA(6))
IF(FORM==2) F1DIARRHOEA = 1

;PPI effect on susp only
IF(PPI==2)  F1PPI = 1  ; Not on PPI
IF(PPI==1)  F1PPI = ( 1 + THETA(7))
IF(FORM==2) F1PPI = 1

;Dose effect on F
DOSF = 0
D50  = 0
IF(FORM==1) D50 = THETA(5)
IF(AMT>0)   DOSE = AMT
DOSEM2  = DOSE / BSA
IF(FORM==1) DOSF  = (DOSEM2 / (DOSEM2 + D50))
TVF1    = (1 - DOSF) * F1DIARRHOEA * F1PPI

F1      = TVF1

K       = CL/VC
S2      = VC
```

```
$ERROR
  CP    = A(2)/S2
  IPRED = CP
  PE    = EPS(1) ; proportional error
  AE    = EPS(2) ; additive error
  Y     = IPRED*(1 + PE) + AE

  PROP  = SQRT(SIGMA(1,1))*IPRED
  ADD   = SQRT(SIGMA(2,2))
  SD    = SQRT(PROP*PROP + ADD*ADD) ; Standard deviation
  IRES  = DV-IPRED
  IWRES = IRES/SD
;
$THETA  (0,14.9512) ; CL
$THETA  (0,201.68) ; VC from Petitcollin et al
$THETA  0.197 FIX ; Ka susp from Ezzet et al
$THETA  0.588 FIX ; Ka tab from Petitcollin et al
$THETA  (0,99.0449) ; D50
$THETA  (-1,-0.332839,5) ; F1DIARRHOEA1
$THETA  (-1,-0.416551,5) ; F1PPI1
;
$OMEGA  0.400179  ;        CL
;
$SIGMA  0.223604
$SIGMA  0.0162301
;;;
$ESTIMATION MAXEVAL=9999 PRINT=5 METHOD=COND INTERACTION
$COVARIANCE PRINT=E
$TABLE      ID TIME EVID IPRED TAD IWRES CWRES NPDE IPRED NOPRINT
            ONEHEADER FILE=sdtab6
$TABLE      ID CL VC KA F1 ETA1 NOPRINT ONEHEADER NOAPPEND FILE=patab6
$TABLE      ID JAC SEX WT MISSLVL MISSDOST MISSDOS MACROLIDES
            ECHINOCANDINS TERB PI CIC FK MMF RIF CBZ PHT H2 PPI ART
            VAL VERAPQUIN MISSFREQ MISSFORM MISSWT DIARRHOEA
            PROPHYLAXIS BLQ NSAMP ONESAMP SUSP NOPRINT ONEHEADER
            NOAPPEND FILE=catab6
$TABLE      ID WT AGE BSA NOPRINT ONEHEADER NOAPPEND FILE=cotab6
```
